# Supplementary material for: Identification of photoperiod-induced specific miRNAs in the adrenal glands of Sunite sheep (Ovis aries)
Source: Front Vet Sci. 2022 Jul 22;9:888207. doi: 10.3389/fvets.2022.888207 (PMC9354845; doi:10.3389/fvets.2022.888207)
Supplement: Supplementary file 1 [file Data_Sheet_1.ZIP › Supplementary file/miRDeep2 file.zip!/miRDeep2 file/Novel_Structure/LP42A3/Novel_108.pdf]

Provisional ID	:	NC_019460.2_5481
Score total	:	2.2
Score for star read(s)	:	-1.3
Score for read counts	:	0
Score for mfe	:	1.9
Score for randfold	:	1.6
Score for cons. seed	:	
Total read count	:	135
Mature read count	:	135
Loop read count	:	0
Star read count	:	0

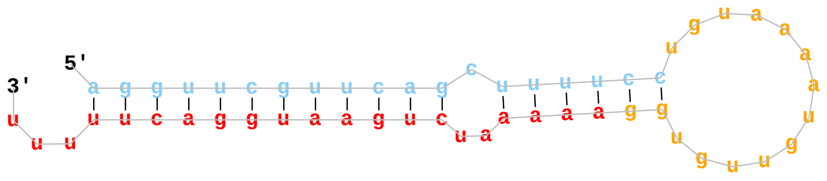

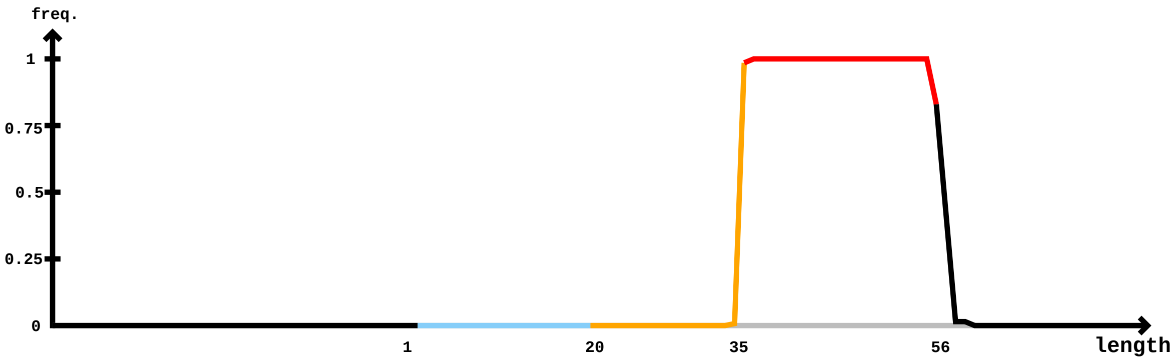


```
                                               Star                               Mature
5'- gaaguaacugcucacagcagauacuggguaggccaaagguucguucagcuuuuccuguaaaauguuguggaaaaaucugaauggacuuuuuggccaacucagcaucucccga   -3'    exp
    ..((((.(((((...))))).))))((((.((((((((((((((((((.((((((.............))))))..)))))))))).)))))))).))))............     reads      mm         sample
    .....................................................................gaaaaaucugaaugAacuuuuu.....................     1          1          seq
    ......................................................................aaaaaucugaaugAacuuuu......................     23         1          seq
    ......................................................................aaaaaCcugaauggacuuuuu.....................     1          1          seq
    ......................................................................aaaaaucugaaugAacuuuuu.....................     65         1          seq
    ......................................................................aaaaaucugaaugAacuuuuug....................     42         1          seq
    ......................................................................aaaaaucugaauggacuuuuug....................     1          0          seq
    .......................................................................aaaaucugaaugAacuuuuuggc..................     2          1          seq
```
